# Predicting recurrence of non-small cell lung cancer based on mean computed tomography value

**DOI:** 10.1186/s13019-021-01476-0

**Published:** 2021-05-12

**Authors:** Masaya Tamura, Isao Matsumoto, Yusuke Tanaka, Daisuke Saito, Shuhei Yoshida, Munehisa Takata

**Affiliations:** Department of Thoracic Surgery, Kanazawa University School of Medicine, Takara-machi 13-1, Kanazawa, 920-8640 Japan

**Keywords:** Ground-glass opacity, Mean- computed tomography value, Recurrence free survival

## Abstract

**Background:**

The aim of this study was to assess the ability of using mean computed tomography (mCT) values to predict non-small cell lung cancer (NSCLC) tumor recurrence.

**Methods:**

A retrospective study was conducted on 494 patients with stage IA NSCLC. Receiver operating characteristics analysis was used to assess the ability to use mCT value, C/T ratio, tumor size, and SUV to predict tumor recurrence. Multiple logistic regression analyses were performed to determine the independent variables for the prediction of tumor recurrence.

**Results:**

The m-CT values were − 213.7 ± 10.2 Hounsfield Units (HU) for the recurrence group and − 594.1 ± 11.6 HU for the non-recurrence group (*p* < 0.0001). Recurrence occurred in 45 patients (9.1%). The tumor recurrence group was strongly associated with a high CT attenuation value, high C/T ratio, large solid tumor size, and SUV. The diagnostic value of mCT value was more accurate than the C/T ratio, excluding the pure ground-glass opacity and pure solid (0 < C/T ratio < 100) groups. The SUV and mCT are independent predictive factors of tumor recurrence.

**Conclusions:**

The evaluation of mCT values was useful for predicting recurrence after the limited resection of small-sized NSCLC, and may potentially contribute to the selection of suitable treatment strategies.

## Background

Ground-glass-opacity (GGO) is defined as a shadow that is completely occupied by a hazy area of increased attenuation in the lung with preserved bronchial and vascular lesion margins when assessed using high resolution computed tomography (HRCT) [1]. Controversy still remains as to the indication of surgical resection or operative procedure for GGO lesions.

It was recently reported that a limited pulmonary resection (wedge resection or segmentectomy) was not inferior to a lobectomy in the management of peripheral small-sized (tumor size ≤20 mm) adenocarcinoma of the lung. The results of the only randomized controlled trial comparing lobectomy with sublobar resection for tumors < 3 cm showed that in comparison to a lobectomy, limited resection was associated with a 75% increase in recurrence rates and a 50% increase in deaths from cancer [2]. Matsumura et al. [3] reported both early and late recurrence after intentional limited resection for cT1aN0M0 non-small cell lung cancer (NSCLC), and reported that of the 21 late recurrence patients, 17(81%) had tumors with a consolidation/tumor ratio (CTR) > 0.25.

Patients with GGO-dominant small lung cancer are believed to have a good prognosis [4, 5]. However, there is no standard method for measuring the area of GGO, and it is not always possible to evaluate the proportion of the solid area in the presence of mixed GGO lesions [6]. Quantitative densitometric methodologies, and mean CT (mCT) number have been reportedly used to evaluate GGO lesions [7–10]. We previously reported that the mCT value of GGO lesions is a risk factor associated with their future change [11], and the evaluation of mCT values is useful in predicting less invasive lung cancer [12]. There have been no studies regarding the use of mCT values in lung cancer and recurrence, which can be of great significance for treatment decisions.

The objectives of this study were to investigate the usefulness of using mCT values for predicting recurrence, and to assess whether they contribute to determining the benefits of performing a limited resection.

## Patients and methods

### Patients

This study was approved by our hospital’s internal review board (2019–137, 3180). Between August 2006 and December 2015, 1413 consecutive patients underwent pulmonary resection for lung cancer.

From these patients, those diagnosed with clinical stage IA lung cancer with a peripherally located tumor were reviewed retrospectively. Lesions with the following criteria were included in this study: radiologically suspicious lung cancer, no sign of nodal involvement, maximum tumor diameter is ≤2 cm, or > 2 cm, ≤3 cm and C/T ratio ≤ 0.5. Patients who received preoperative treatment, such as radiotherapy or chemotherapy, or had multiple lung cancers were excluded from the study.

A total of 494 patients met both clinical and imaging criteria for inclusion in this study. Of these, 228 were men and 266 were women. Their ages ranged from 19 to 91 years, with a median of 66 years. We reviewed their medical records, including the results of pathologic examination and recurrence status. For each case, the surgical specimens were reviewed and classified according to the latest 2015 WHO classification criteria for lung adenocarcinoma as AAH, AIS, and MIA [13].

### Patient follow-up and outcomes

The median follow-up time frame of patients was 55 months (mean: 58.5; 24-150 months). Routine follow-up included a physical examination, chest CT scan, and blood test every 3–6 months for the first 3 years after surgery and every 6–12 months thereafter. Magnetic resonance imaging and 18F-fluorodeoxyglucose positron emission tomography were also performed as necessary. The primary endpoint was recurrence-free survival, measured from the date of the initial surgical resection to the first evidence of tumor recurrence. Recurrence was diagnosed by physical examination and diagnostic imaging of the lesions. Diagnosis was also histologically confirmed when clinically feasible. Second primary tumors were defined using all available radiologic and pathologic information according to a modified version of the criteria of Martini and Meland [14]. We defined them as a new pulmonary malignancy occurring in a different lobe or lung than the first tumor with no intervening lymph nodes and no evidence of metastases, different histology or subtype and/or molecular genomic differences.

### Image acquisition and analysis

CT scans were performed from lung apex to base at mid-inspiration during a held breath using a section thickness of 1.25 or 2.5 mm (Asteion 4, Toshiba, Tokyo, Japan). Two radiologists with 20 and 15 years of experience independently viewed these images and subjectively classified the nodules. Pure GGO was defined as a shadow that was completely occupied by a hazy area of increased attenuation of the lung, with preserved bronchial and vascular margins of the lesion with no solid regions on HRCT. The longest diameters of the GGO lesions and solid portion were measured. The proportion of GGO was calculated using a previously published method [15] and defined as the C/T ratio. The maximum diameter and one-dimensional mCT values were measured using a computer graphics support system (Synapse® PACS, Fujifilm, Tokyo, Japan). The shape of the region of interest was standardized for each patient and configured by freehand drawing. The mCT value was evaluated in slice having highest density. The multi-observer variation was corrected by calculating the mean value from the two observers.

### Statistical analysis

Receiver operating characteristics (ROC) analysis was used to compare the ability to predict the recurrence of the lung cancer using the mCT value, SUV value, C/T ratio, solid tumor size, and carcinoembryonic antigen (CEA) cancer blood test value. Univariate and multivariate analysis were carried out to investigate potential pretreatment predictors of recurrence. Univariate and multivariate Cox proportional hazard regression analyses were conducted to evaluate the associations between clinical and imaging metrics with clinical outcome. The 95% confidence interval (95% CI) was calculated and all *p-*values were two-sided. Gender, age, type of resection, tumor size, CEA value, SUV, C/T ratio, mCT value, solid tumor size were all included in the univariate analysis. Since this was a retrospective study, the variables for univariate analysis were selected postoperatively. Variables that can be assessed from medical records, radiologic imaging, nuclear medicine examination, and blood tests that are useful for diagnosing malignant potential were included in the univariate analysis. Univariate factors with a *p*-value of < 0.05 were included in the multivariate analysis. All data regarding continuous variables were expressed as mean ± SD. Significant differences were assessed using the *t*-test for continuous variables and the chi-square -test for categorical variables. Analyses were performed using the SAS software package (SAS Institute, Inc., Cary, NC). A *p*-value of < 0.05 was considered statistically significant.

## Results

**Fig. 1:**
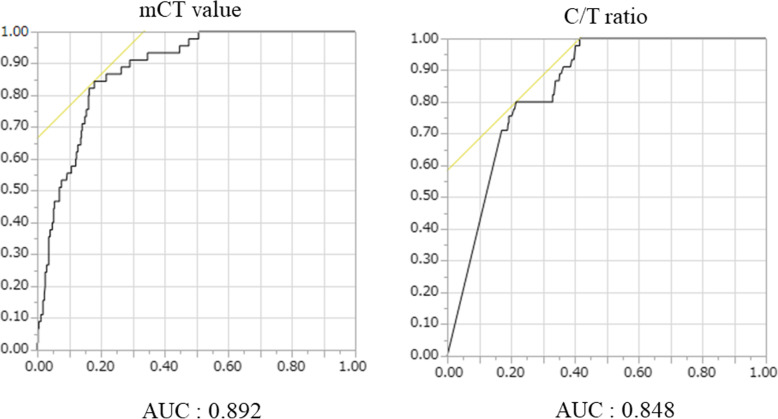
Receiver operating characteristics curves predicting the tumor recurrence. left: mCT value, right: C/T ratio, (mCT value = mean computed tomography value, C/T ratio = consolidation/tumor ratio)

The CT findings of typical GGO lesions are presented in the Fig. 1 and detailed explanation were described in the results section of the report already published 12. The mCT values were − 213.7 ± 10.2 HU for the recurrence group and − 594.1 ± 11.6 HU for the non-recurrence group (*p* < 0.0001). Clinical and pathological characteristics are shown in Table 1. During the study period, recurrence occurred in 45 patients (9.1%): 3 (6.7%) were stump recurrence, 9 (20%) were in a different lung lobe, 8 (17.8%) were in a lymph node, 6 (13.3%) were dissemination, and 19 (42.2%) were distant metastasis. Thirty-one (68.9%) out of 45 cases were diagnosed histologically. Fourteen cases were diagnosed by CT guided biopsy, 9 cases were surgery and 8 were by TBLB or TBNA.

**Table 1:** Clinical and pathological characteristics

Factors	Number	%
Gender		
Male	228	46.2
Female	266	53.8
Age		
Range	19 - 91	
Mean±SD	65.3±11.3	
Clinical T stage		
Tis	217	43.9
T1mi	57	11.5
T1a	83	16.8
T1b	137	27.8
IASLC		
AAH	7	1.4
AIS	112	22.7
MIA	208	42.1
Invasive adenocarcinoma	167	33.8
Histology		
Adenocarcinoma	452	91.5
Squamous cell carcinoma	33	6.7
Otheors	9	1.8
Operative procedure		
Partial resection	276	55.9
Segmentectomy	218	44.1

*IASLC* The international association for the study of lung cancer, *AAH* Atypical adenomatous hyperplasia, *AIS* Adenocarcinoma in situ, *MIA* Minimally invasive adenocarcinoma

The comparison of clinico-radiological data between lesions in recurrence and non-recurrence are summarized in Table 2. The recurrence group was strongly associated with a high mCT value, high C/T ratio, large solid tumor size, and high SUV. Age, CEA, SUV, C/T ratio, solid tumor size, and mCT value were selected factors for the multivariate analysis based on a *p*-value cutoff and possibly based on recognized clinical parameters. We attempted to predict which cancer would recur based on the mCT value, SUV, C/T ratio, solid tumor size, and CEA, and ROC curve analysis was performed to determine the appropriate cutoff value (Fig. 1). The maximum sensitivity and specificity were obtained at a cutoff value of − 506 HU, 2.5, 38.2%, 0 mm, and 5.0, respectively. The ROC area under the curve value of the mCT value was the highest (0.892; 95% CI: 0.79–0.91), followed by SUV (0.864; 95% CI: 0.76–0.88), C/T ratio (0.848; 95% CI: 0.79–0.86), solid tumor size (0.802, 95% CI: 0.69–0.84), and CEA (0.763, 95% CI: 0.71–0.82). In the patients with non-adenocarcinoma and the pure solid group, the diagnostic value of mCT value or SUV was poorer compared to that of the adenocarcinoma group. The diagnostic value of mCT value was superior to the C/T ratio, excluding the pure GGO and pure solid (i.e. 0 < C/T ratio < 100) groups (Table 3). Table 4 shows the results of univariate and multivariate analyses for predicting tumor recurrence. Multiple logistic regression analyses using the preoperatively determined variables revealed that SUV (*p* = 0.041) and mCT values (*p* = 0.029) are independent predictive factors of recurrence. However, the C/T ratio was not significant (*p* = 0.19).

**Table 2:** Comparison of clinico-radiological data between lesions in recurrence(+) and recurrence(-) categories

Factors	Recurrence(-) (n)	Recurrence(+) (n)	***p*** value
Gender			<0.0001
Male	195	33	
Female	254	12	
Age			<0.0001
<65 years	207	6	
≥65 years	242	39	
Lesion size			<0.0001
<12.9 mm	249	6	
≥12.9 mm	200	39	
Histology			<0.0001
Non adenoca	29	13	
Adenoca	420	32	
mCT value (H.U)			<0.0001
< -506	162	43	
≥-506	287	2	
C/T ratio			<0.0001
< 38.2	178	39	
>38.2	271	6	
SUV			<0.0001
< 2.5	426	41	
>2.5	23	4	
CEA			0.0022
< 5.0	410	27	
≥5.0	39	18	
Operative procedure			0.122
Partial resection	246	30	
Segmentectomy	203	15	
Solid tumor size (mm)			<0.0001
0	217	0	
> 0	232	45	

*n* number of cases, *non-recurrence (-)* without recurrence, *recurrence (+)* with recurrence, *HU* Hounsfield unit, *CT* Computed tomography, *C/T ratio* Consolidation / tumor ratio, *SUV* Standardized uptake value, *CEA* Carcinoembryonic antigen

**Table 3:** Comparison of AUC between m-CT value, SUV value, C/T ratio and CEA

Factors	Total	Adeno	Non-adeno	Pure solid	0<CT ratio <100
mean CT value	0.892	0.879	0.791	0.662	0.762
SUV value	0.864	0.884	0.55	0.607	0.847
C/T ratio	0.848	0.856	-	-	0.571
CEA	0.763	0.771	0.47	0.532	0.512

*CT* Computed tomography, *SUV* Standardized uptake value, *C/T ratio* Consolidation / tumor ratio, *CEA* Carcinoembryonic antigen

**Table 4:** Multiple logistic regression analysis predicting the tumor recurrence

		Univariate analysis				Multivariate analysis		
Risk factor	HR	95% CI		***P-***value	HR	95% CI		***P-***value
Age								
65< vs ≦65	1.75	1.17	2.73	0.055				
Type of resection								
PR vs Seg or Lob	1.31	0.944	1.81	0.11				
Tumor size								
12.9 < vs ≦12.9	2.07	1.53	2.90	0.0001	1.28	0.91	2.21	0.26
CEA								
5.0< vs ≦5.0	1.36	0.667	2.33	0.35				
SUVmax value								
2.5< vs ≦2.5	2.36	1.57	3.77	<0.0001	2.12	1.12	2.68	0.058
C/T ratio								
38.2 < vs ≦38.2	2.96	2.01	4.80	<0.0001	1.38	0.86	2.99	0.20
mCT value (H.U)								
≦-506 vs -506 >	3.41	2.31	5.52	<0.0001	2.92	1.29	11.2	0.043
Solid size								
0 < vs 0	3.93	2.59	6.68	<0.0001	1.49	0.78	7.12	0.11
Ly(-) and v(-) vs others	3.56	2.21	4.48	<0.0001	3.12	1.41	5.57	0.039

*PR* Partial resection, *Seg* Segmentectomy, *CEA* Carcinoembryonic antigen, *SUV* Standardized uptake value, *C/T ratio* Consolidation / tumor ratio, *mCT* Mean computed tomography, *HU* Hounsfield unit

## Discussion

The present study aimed to identify the risk factors associated with tumor recurrence after limited resection for small-sized NSCLC. Evaluation of various CT features, such as the mCT values, C/T ratio, solid tumor size, whole tumor size, and SUV can be helpful in predicting the potential for tumor recurrence. In particular, we initially demonstrated that the mCT value of the GGO lesion is a sensitive marker for predicting tumor recurrence.

Although lymphatic or blood vessel invasion is reportedly a strong predictor for postoperative tumor recurrence in completely resected stage I non-small cell carcinoma [16–19], it is difficult to preoperatively identify patients with stage I adenocarcinoma who will have lymphatic or vessel invasion. The preoperative ability to biologically distinguish aggressive tumors from indolent tumors is extremely important for judging whether patients are suitable for sublobar resection. A considerable effort has been exerted to preoperatively distinguish non-invasive versus invasive cancer. In the clinical setting, pathological examination was more important when selecting cases for limited resection. We previously reported that the evaluation of mCT value is useful in predicting less invasive lung cancer [12]. There have been no studies regarding the mCT value in lung cancer and recurrence, which is of great significance for treatment decisions. In the present study, we had selected tumor recurrence as the endpoint.

With recent advances in diagnostic imaging technologies, GGO lesions are increasingly detected using HRCT scans [20, 21]. GGO is defined as a shadow that is completely occupied by a hazy area of increased attenuation in the lung with preserved bronchial and vascular lesion margins when assessed using HRCT [1]. In a clinical setting, several types of GGO can be encountered. It is difficult to measure the size of the solid part of the tumor when the nodule comprises a heterogeneous mixture of GGO and solid tumor. Suzuki et al. [6] classified peripheral small-sized adenocarcinoma into six categories, and reported that the classification was significantly associated with pathologic prognostic factors.

Several authors have classified small lung lesions into nonsolid (pure) GGO, partly solid (mixed) GGO, and solid types. However, it is sometimes difficult to differentiate between pure and mixed GGO, and between high-density GGO and solid tumors, because no definite radiological criteria exist to distinguish these differences. Some authors used quantitative densitometric methodologies to evaluate GGO lesions [11, 19–21]. Although the one-dimensional quantitative mCT value can be slightly affected by the densities of vessels or bronchi within the tumor, this calculation method is straightforward, and can similarly estimate pure GGO and mixed GGO.

Our study indicated that the predictive ability of the mCT value for tumor recurrence was higher for small-sized tumors and tumors showing extensive GGO. For solid-predominant tumors, SUV demonstrated higher predictive ability than did the mCT value. The mCT value is useful for tumors that are mainly homogeneous in density, or those that are too dense to be called pure GGO but are not pure solid tumor. The mCT value adds a diagnostic value to the C/T ratio for tumors for which the size of solid portion is difficult to measure. The ROC area under the curve value of the mCT value was the highest, excluding the pure GGO and pure solid (i.e. 0 < C/T ratio < 100) group (Table 3). The GGO legion is commonly seen with adenocarcinomas, while most squamous cell carcinomas or other histology groups show pure solid lesion. That is the reason why the diagnostic power of mCT, SUV, and C/T ratio is seen in the adenocarcinoma group.

This study has several limitations. First, it was performed retrospectively. Second, the cut-off values of the ROC curves that dichotomized the two groups could be an arbitrary value and not available universally. Fourth, the follow-up duration was rather short, even though the mean interval between the two groups, with and without recurrence, showed no differences (*p* = 0.52).

## Conclusions

In conclusion, mCT values were useful for predicting recurrence after the limited resection of small-sized NSCLC, in particular, such as patients with adenocarcinoma manifested as mixed GGO, added new information to the C/T ratio, and may contribute to selecting suitable treatment strategies. A future prospective study should be conducted to establish the optimal treatment strategies for this disease.

## Data Availability

Not applicable.

## Abbreviations

AAH: Atypical adenomatous hyperplasia
AIS: Adenocarcinoma in situ
C/T ratio: Consolidation / tumor ratio
DFS: Disease-free survival
GGO: Ground-glass opacity
HRCT: High-resolution computed tomography
HU: Housfield units
m-CT value: Mean computed tomography value
MIA: Minimally invasive adenocarcinoma
ROC: Receiver operating characteristics
SUV: Standardized uptake value
